# Supplementary material for: Bioinformatics Analysis of the Prognostic Significance of CAND1 in ERα-Positive Breast Cancer
Source: Diagnostics (Basel). 2022 Sep 27;12(10):2327. doi: 10.3390/diagnostics12102327 (PMC9600875; doi:10.3390/diagnostics12102327)
Supplement: Supplementary file 1 [file diagnostics-12-02327-s001.zip › Table S2.pdf]

Table S2. List of miRNAs that target CAND1

microRNA that targets CAND1	Function	Reference
miR-30d-5p	Sensitizes ovarian cancer cells to apoptosis by supressing PI3KT/AKT pathway	[53]
miR-33a	Decreases lung cancer proliferation and migration by negatively regulating CAND1	[14], [54]
miR-148b-3p	Increases Schwann cells migration by negatively regulating CAND1	[17]
miR-933	Inhibits proliferation and invasion of lung cancer	[55]
