# Supplementary figures and images for: Bioinformatics Analysis of the Prognostic Significance of CAND1 in ERα-Positive Breast Cancer
Source: Diagnostics (Basel). 2022 Sep 27;12(10):2327. doi: 10.3390/diagnostics12102327 (PMC9600875; doi:10.3390/diagnostics12102327)

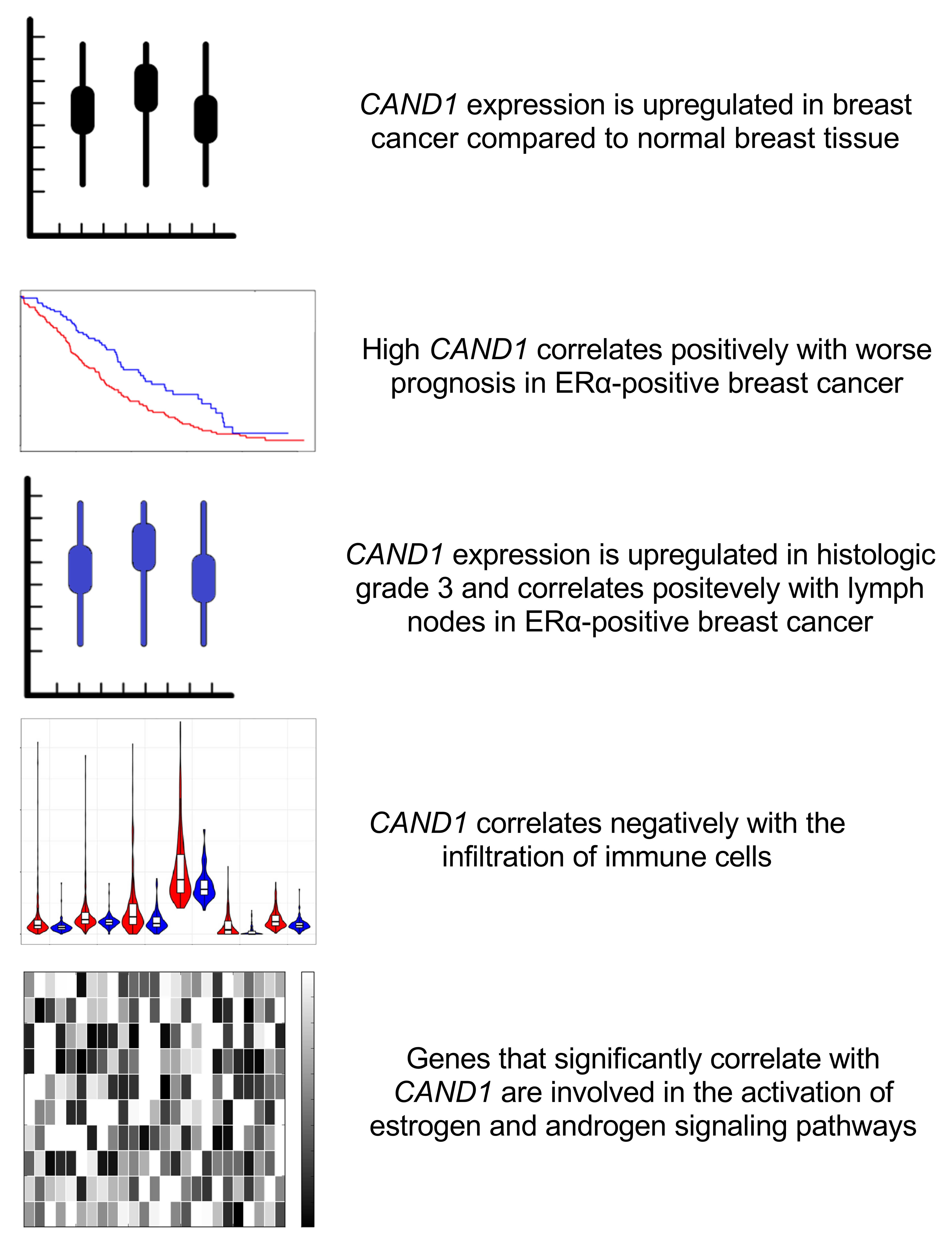

Supplement: Supplementary file 1 [file diagnostics-12-02327-s001.zip › Figure S1.tif]
